# Percutaneous endoscopic transforaminal discectomy precedes interlaminar discectomy in the efficacy and safety for lumbar disc herniation

**DOI:** 10.1042/BSR20181866

**Published:** 2019-02-15

**Authors:** Peng Chen, Yihe Hu, Zhanzhan Li

**Affiliations:** 1 Department of Orthopedic, Xiangya Hospital, Central South University, Changsha, Hunan Province, China; 2 Department of Oncology, Xiangya Hospital, Central South University, Changsha, Hunan Province, China

**Keywords:** Lumbar disc herniation, microendoscopic discectomy, meta-analysis, percutaneous endoscopic lumbar discectomy

## Abstract

We searched several databases from the times of their inception to 20 December 2018. Randomized controlled trials and cohort studies that compared percutaneous endoscopic transforaminal discectomy (PETD) with percutaneous endoscopic interlaminar discectomy (PEID) were identified. We used a random-effects model to calculate the relative risks (RRs) of, and standardized mean differences (SMDs) between the two techniques, with 95% confidence intervals (CIs). Twenty-six studies with 3294 patients were included in the final analysis. Compared with PEID, PETD reduced the short-term (SMD −0.68; 95% CI −1.01, −0.34; *P*=0.000) and long-term (SMD −0.47; 95% CI −0.82, −0.12; *P*=0.000) visual analog scale scores, blood loss (SMD −4.75; 95% CI −5.80, −3.71; *P*=0.000), duration of hospital stay (SMD −1.86; 95% CI −2.36, −1.37; *P*=0.000), and length of incision (SMD −3.93; 95% CI −5.23, −2.62; *P*=0.000). However, PEID was associated with a lower recurrence rate (*P*=0.035) and a shorter operative time (*P*=0.014). PETD and PEID afforded comparable excellent- and good-quality data, long- and short-term Oswestry disability index (ODI) scores, and complication rates. PETD treated lumbar disc herniation (LDH) more effectively than PEID. Although PETD required a longer operative time, PETD was as safe as PEID, and was associated with less blood loss, a shorter hospital stay, and a shorter incision. PETD is the best option for patients with LDH.

## Introduction

Lumbar disc herniation (LDH) is common today, even in young individuals, and more often in males than females [1]. Most herniation sites are located at L5/S1 and L4/5. LDH is caused by degenerative changes in the intervertebral discs; external forces cause rupture of the annulus fibrosus, nuclear herniation, or compression of the cauda equina nerve roots, triggering tissue inflammation, edema, and poor microcirculation, followed by low back pain, lower extremity sciatic radiating pain, and other disorders [2], in turn compromising the quality of life [3]. Therapeutic strategies include conservative and surgical treatments. Most patients benefit greatly from conservative treatments, such as intravenous and oral medication administration, traction therapy, and manipulative rehabilitation, but some require surgery [4]. The surgical options include open discectomy, microdiscectomy, microendoscopic discectomy (MED), and percutaneous endoscopy lumbar discectomy (PELD) [5]. In recent years, with the rapid development of surgical techniques, minimally invasive spine surgery has become imperative. Compared with open discectomy, minimally invasive surgery is associated with a shorter operative time, less blood loss, less muscle injury, and faster functional recovery [6–8]. PELD includes percutaneous endoscopic transforaminal discectomy (PETD) and percutaneous endoscopic interlaminar discectomy (PEID). Some previous studies confirmed the therapeutic efficacy of PETD, but others did not [9,10]. PETD is rather difficult in patients with high cristae iliacae and narrow foramina, especially at L5/S1. However, the L5/S1 interlaminar space is usually adequate [11]. Ruetten et al. [12] were the first to perform intervertebral disc discectomy and decompression by creating an intervertebral foramen in the vertebral canal between the upper and lower vertebral discs. Several studies have compared the efficacies of PETD and PEID in patients with LDH; the results were inconsistent. Hence, we comprehensively analyzed this topic.

## Materials and methods

### Search strategy

Two investigators (P.C. and Z.L.) independently searched the following databases from their inception to 20 July 2018: PubMed, Web of Science, Embase, China National Knowledge Infrastructure, and WanFang. The following search terms were used: ‘lumbar disc herniation’ OR ‘LDH’, ‘percutaneous endoscopic lumbar discectomy’ OR ‘percutaneous endoscopic transforaminal discectomy’ OR ‘PLED’ OR ‘PELD’, and ‘microendoscopic discectomy’ OR ‘percutaneous endoscopic interlaminar discectomy’. We restricted the languages to Chinese and English. We checked the reference lists of selected full-text and review articles to identify other potentially relevant works.

### Inclusion and exclusion criteria

The inclusion criteria were: (i) examination of a population of patients with LDH, (ii) randomized controlled trial or retrospective study comparing the efficacies of PETD and PEID in terms of LDH treatment, and (iii) comparison of PETD and PEID interventions. The primary outcome requirements were: (i) at least one short-term or long-term visual analog scale (VAS) or Oswestry disability index (ODI) score, and (ii) excellent or good data quality. The secondary outcomes were the (iii) complication rate, (iv) recurrence rate, (v) operative duration, (vi) amount of blood loss, (vii) length of incision, and (viii) length of hospital stay. Reviews, comments, duplicate and case reports, letters, and animal and experimental studies were excluded.

### Data extraction and quality assessment

We used a standard Excel sheet for data extraction. Two investigators (P.C. and Z.L.) independently extracted the following data: first author, publication year, mean patient age, study design (retrospective compared with prospective), sample size, follow-up duration, and outcome measures. We sought to contact the authors when information was missing. Differences in opinion were resolved by discussion with the third investigator (Y.H.).

All prospective and retrospective studies were evaluated using the Newcastle–Ottawa scale (which compares patient selection, comparisons, and outcomes; maximal score 9). Studies with scores ≥7 were considered to be of high quality [13]. We used the Cochrane risk-of-bias tool to assess study quality [14]; the tool explores random sequencing, allocation concealment, blinding of participants and personnel, outcome assessments, outcome data completeness, selective reporting, and other biases. We scored each study as being at a low, high, or unclear risk of bias. Studies in which at least one key domain was considered to be at high risk of bias were regarded as high risk; other studies were considered to be of low or unclear risk.

### Statistical analysis

We used fixed- and random-effects models to evaluate pooled data [15]. We calculated relative risks (RRs) with 95% confidence intervals (CIs) for dichotomous data and standardized mean differences (SMDs) with 95% CIs for continuous data. Within-study heterogeneity was assessed by calculating the *I*^2^ statistic and Cochran’s *Q*; when *I*^2^-values >50% and *P*-values <0.05 indicated significant heterogeneity, we employed the random-effects model [16]; we used the fixed-effects model otherwise. To evaluate heterogeneity further, we performed subgroup analyses of primary outcomes (VAS and ODI scores). Publication bias was assessed by visual inspection of a funnel plot and application of the Egger’s/Begg’s test [17,18]. All statistical analyses were performed with the aid of Stata ver. 14.0 (StataCorp LP) and RevMan ver. 5.3 (Nordic Cochrane Center) software; *P*-values <0.05 were considered to reflect significance.

## Results

### Study selection and characteristics

**Figure 1:**
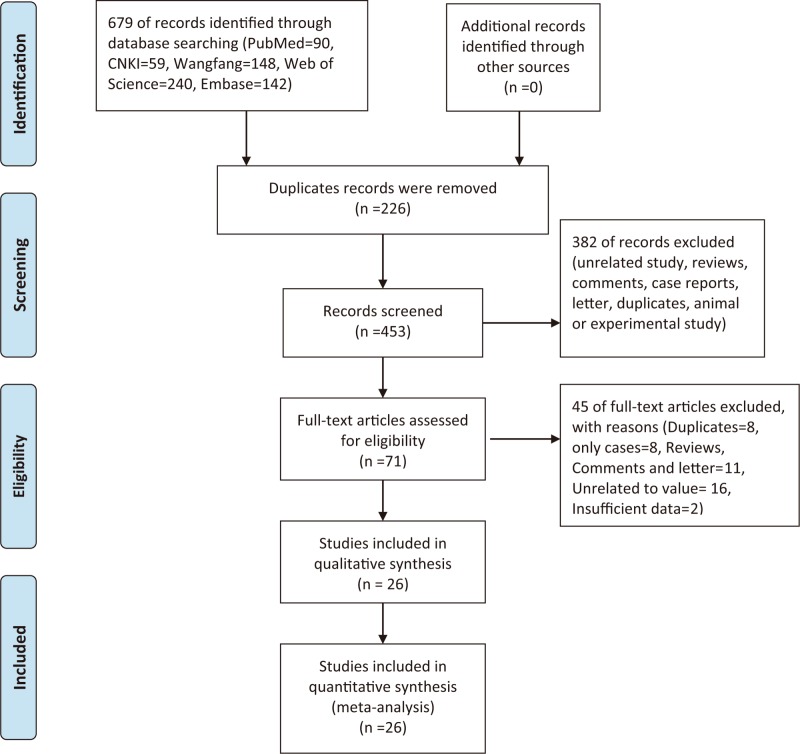
Flow chart of study selection

Figure 1 shows the study selection flow. Our initial search returned 679 records; we found no additional text when exploring other possible sources. After removal of duplicates and scanning of titles and abstracts, we selected 71 full-text articles for further assessment. We excluded 45 of these articles. Finally, 26 studies were included in our qualitative and quantitative analyses (Supplementary Material S1). The general characteristics of the studies are listed in Table 1; all studies were published between 2009 and 2018. The mean ages of patients treated with PETD ranged from 33.1 to 69.2 years, and those of patients treated with PEID ranged from 36.8 to 68.9 years. Nine studies were retrospective and seventeen were prospective. The duration of follow-up ranged from 3 to 26 months. The types of disease included central, paracentral, and far-lateral disease, and disease of the intervertebral foramen.

**Table 1:** Characteristics of included studies in the meta-analysis

Author	Publication year	Age (PEID/PETD)	Study design	Sample size		Follow-up time (months)	Type
				PEID	PETD		
Fang	2012	43.5/45.8	Retrospective	40	40	6	(1)(2)(3)
Le	2014	37.2/38.4	Prospective	190	185	12	(1)(2)(3)
Guan	2014	-	Prospective	35	35	3	-
Wu	2009	4.5/45.8	Prospective	30	30	6	(1)(2)(3)
Wu	2015	38.5/41.3	Prospective	50	36	6	(1)(2)(3)
Zhang	2015	43.2/41.5	Prospective	30	30	26	(3)(4)
Zhang	2015	37.5/35.8	Retrospective	21	21	12	(1)(2)(4)
Fu	2014	-	Prospective	8	62	12	(1)(2)(3)(4)
Zeng	2015	-	Prospective	25	25	24	-
Li	2013	38.3/43.3	Prospective	212	208	-	-
Li	2015	51.5/51.6	Retrospective	50	50	-	-
Yang	2015	48.4/48.0	Prospective	82	57	3	-
Tang	2015	-	Prospective	38	38	24	-
Zhao	2012	39.4/43.2	Retrospective	245	261	-	-
Chen	2015	-	Prospective	25	13	13.5	(3)(4)
Mao	2015	37.5/37.8	Retrospective	35	30	12	-
Yoon	2012	45.9/56.5	Retrospective	37	35	6	-
Sinkemani	2015	44.2/41.5	Retrospective	50	36	14	-
Liu	2012	-	Prospective	25	13	13.5	(3)(4)
Chen	2018	64.1/64.2	Prospective	137	136	12	(1)(2)(4)
Chen	2018	40.7/40.2	Prospective	73	80	12	(1)(2)(3)(4)
Huang	2018	32.1/32.3	Retrospective	52	50	6	(1)(2)(3)(4)
Ding	2017	54.2/54.4	Prospective	40	40	3	(1)(2)(3)(4)
Liu	2017	69.2/68.9	Prospective	25	25	3	(1)(2)(3)(4)
Liu	2018	33.1/36.2	Prospective	63	60	24	(1)(2)(3)(4)
Wu	2017	38.7/40.8	Retrospective	40	40	12	(1)(2)(3)

(1) Central type, (2) Para central, (3) Intervertebral foramen, and (4) Far-lateral.

### Quality assessment

We included 8 randomized controlled trials and 18 follow-up studies. Supplementary Material S2 lists the risks of bias and includes the bias graphs. Two studies were considered to exhibit high risks of bias because neither the study participants nor personnel were blinded. On the Newcastle–Ottawa scale, the mean score of observational studies was >7, indicating high quality.

### Pooled results

The summarized results are presented in Table 2.

**Table 2:** Comparison of pooled parameters between percutaneous endoscopic lumbar, transforaminal discectomy and interlaminar discectomy

Parameters	Number of study	*P*_heterogeneity_	RR/SMD	95% CI	*P*	Egger	Begg
Short-term VAS	12	0.000	−0.68	−1.01, −0.34	0.000	0.012	0.002
Long-term VAS	11	0.000	−0.47	−0.82, −0.12	0.000	0.900	0.224
Short-term ODI	5	0.000	−0.06	−0.33, 0.22	0.691	0.306	0.951
Long-term ODI	7	0.000	−0.15	−0.36, 0.06	0.123	0.238	0.537
Excellent and good rate	13	0.015	1.02	0.97, 1.07	0.509	0.232	0.272
Complication rate	15	0.438	0.78	0.54, 1.13	0.185	0.149	0.400
Recurrence rate	11	0.128	1.90	1.04, 3.47	0.035	0.017	0.008
Duration of operation	18	0.000	0.70	0.14, 1.26	0.014	0.226	0.058
Blood loss	15	0.000	−4.75	−5.80, −3.71	0.000	0.273	0.235
Length of incision	8	0.000	−3.93	−5.23, −2.62	0.000	0.067	0.063
Duration of hospital stay	15	0.000	−1.86	−2.36, −1.37	0.000	0.081	0.038

### Short- and long-term VAS scores

**Figure 2:**
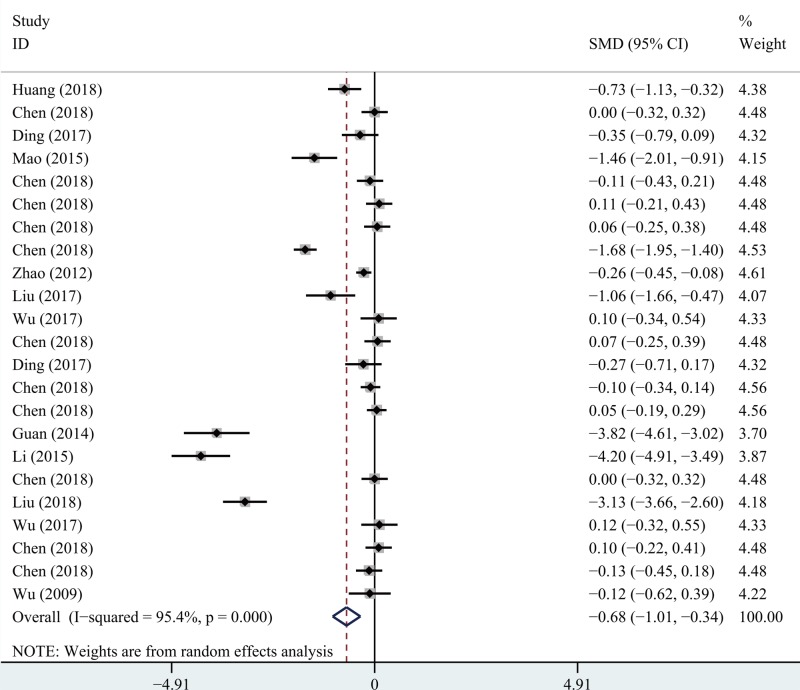
Comparison of short-term VAS between PETD and PEID

**Figure 3:**
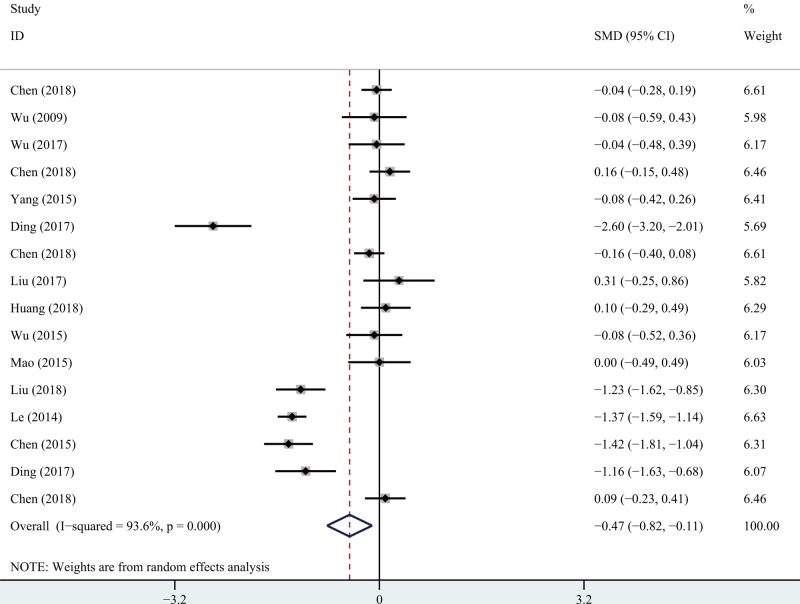
Comparison of long-term VAS between PETD and PEID.

Twelve articles provided short-term and eleven provided long-term VAS scores. Significant heterogeneity was apparent (*I*^2^ > 50%, *P*=0.000). The random-effects model was used to analyze both datasets. Meta-analysis showed that the short-term (SMD −0.68; 95% CI −1.01, −0.34; *P*=0.000; Figure 2 and long-term (SMD −0.47; 95% CI −0.82, −0.12; *P*=0.000; Figure 3) scores associated with PETD were significantly lower than those associated with PEID.

### Short-term and long-term ODI scores

**Figure 4:**
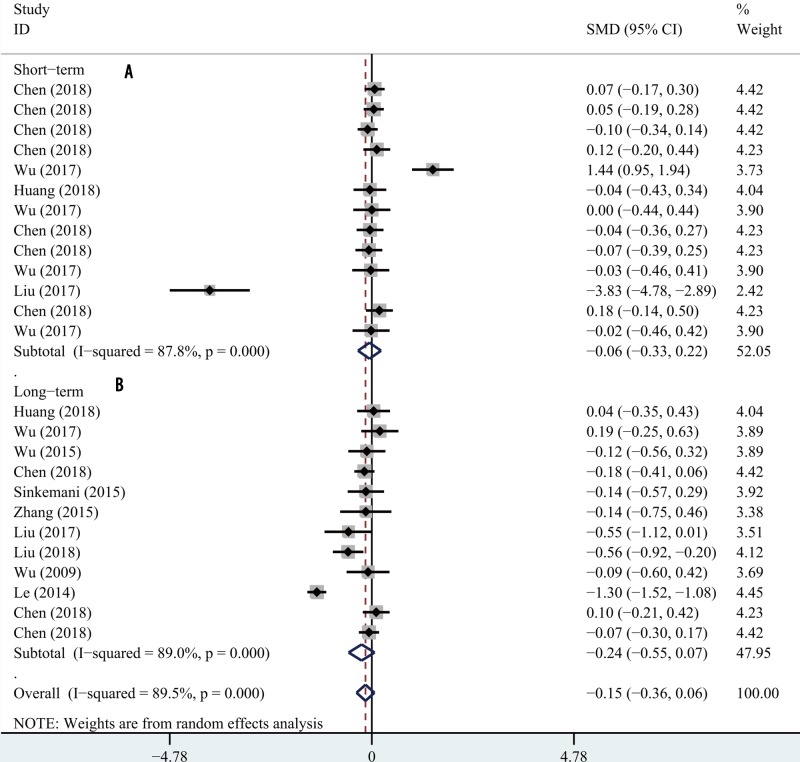
Forest plot for short-term and long-term ODI between PETD and PEID Comparison of short-term (**A**) and long-term (**B**) ODI between PETD and PEID.

Five articles provided short-term ODI scores and seven provided long-term scores. We used a random-effects model for analysis because significant heterogeneity was in play. Neither the short- nor long-term ODI score differed significantly between PETD and PEID (SMD −0.06; 95% CI −0.03, 0.22; *P*=0.691; Figure 4A; and SMD −0.15; 95% CI −0.36, 0.06; *I* = 0.123; Figure 4B, respectively).

### Excellent and good data

**Figure 5:**
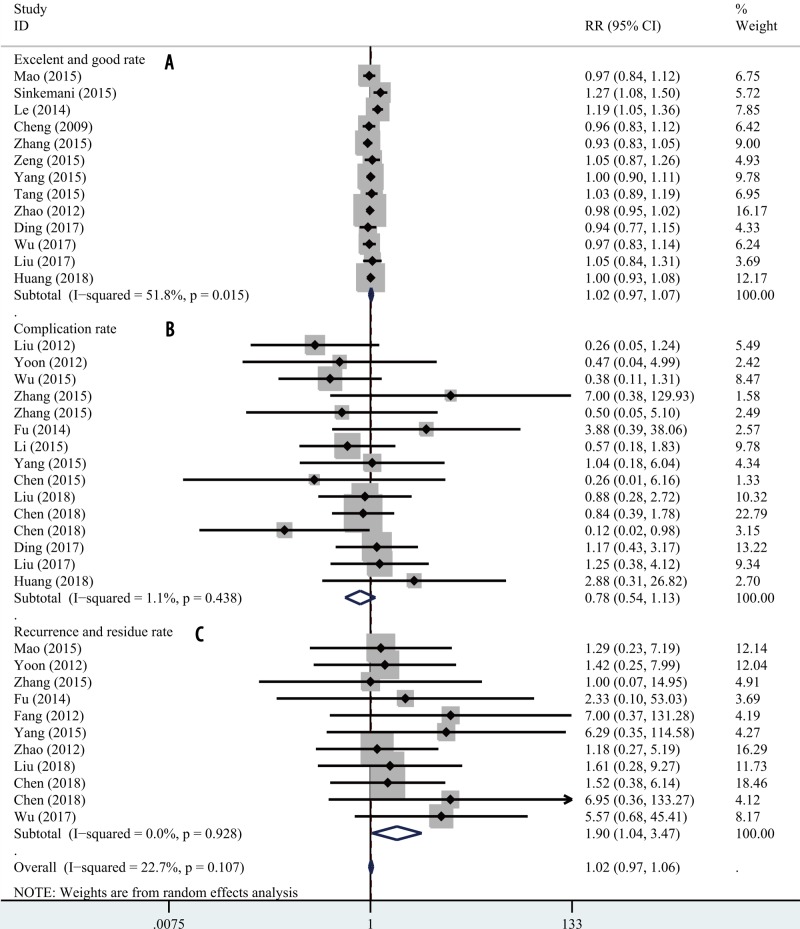
Forest plot for clinical outcomes Comparisons of clinical outcomes between PETD and PEID: (**A**) excellent and good rate; (**B**) complication rate; (**C**) recurrence and residue rate.

The data from 13 studies were rated as excellent or good; the degree of heterogeneity was moderate (*I*^2^ = 51.8%, *P*=0.015). The random-effects model indicated that the excellent and good rates in the two groups were nearly identical (RR = 1.02; 95% CI 0.97, 1.07; *P*=0.509; Figure 5A).

### Complication and recurrence rates

Complication rates were reported in 15 articles; the degree of heterogeneity was very low (*I*^2^ = 1.1%, *P*=0.438). A fixed-effects model revealed no significant between-group difference (RR = 0.78; 95% CI 0.54, 1.13; *P*=0.185; Figure 5B). Recurrence rates were reported in 11 articles; no heterogeneity was evident (*I*^2^ = 0.0%, *P*=0.128) and the data were analyzed using a fixed-effects model. The pooled results suggested that the recurrence rate after PETD was higher than that after PEID (RR = 1.90; 95% CI 1.04, 3.47; *P*=0.035; Figure 5C).

### Duration of operation and blood loss

**Figure 6:**
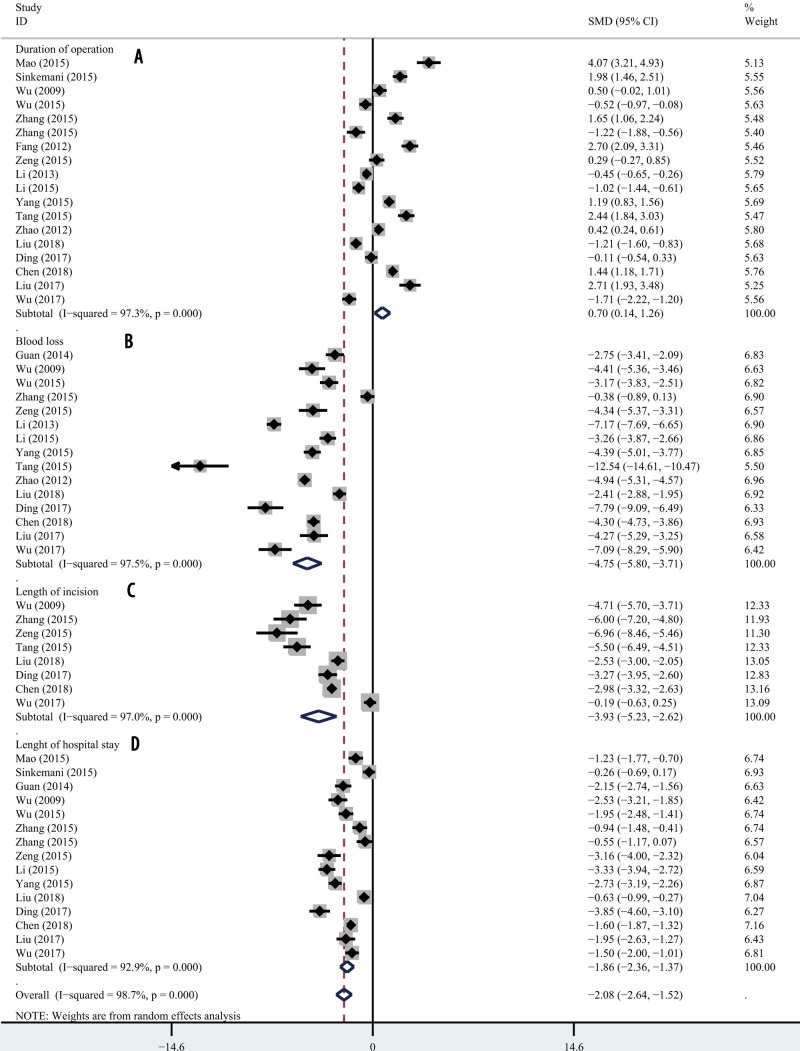
Forest plot for symptoms Comparisons of duration of operation (**A**), blood loss (**B**), length of incision (**C**), and length of hospital stay (**D**).

Random-effects models were used to analyze the duration of operation and blood loss because significant heterogeneity was evident (*I*^2^ > 50.0%, *P*<0.05). Eighteen articles provided data on the operative duration and fifteen provided data on blood loss. Compared with PEID, PETD required a longer operative time (SMD 0.70; 95% CI 0.14, 1.26; *P*=0.014; Figure 6A), but was associated with less blood loss (SMD −4.75; 95% CI −5.80, −3.71; *P*=0.000; Figure 6B).

### Length of incision and duration of hospital stay

The length of incision and duration of hospital stay were also evaluated. Eight articles provided data on the length of incision and fifteen provided data on the duration of hospital stay. Both indicators evidenced significant heterogeneity (*I*^2^ > 50.0%, *P*<0.0.5). Random-effects models indicated that PETD required a shorter incision (SMD −3.93; 95% CI −5.23, −2.62; *P*=0.000; Figure 6C) and a shorter hospital stay (SMD −1.86; 95% CI −2.36, −1.37; *P*=0.000; Figure 6D).

### Sensitivity analysis and publication bias

We subjected the operative durations reported in most (*n*=18) articles to sensitivity analysis; we omitted one study at a time. The pooled results ranged from 0.10 to 0.63 (Supplementary Material S3). All results were significant. The Egger’s/Begg’s test indicated that publication bias was not in play (*P*>0.05), except in terms of the short-term VAS score and the recurrence rate (Table 2). The funnel plot was slightly asymmetrical (Supplementary Material S4).

## Discussion

We comprehensively and systematically reviewed the literature and found that: (i) PETD resulted in lower short- and long-term VAS scores than PEID, despite the absence of a significant difference between PETD and PEID in terms of short- and long-term ODI scores and the numbers of studies of excellent and good quality; (ii) although the complication rates of PETD and PEID were similar, PEID was associated with significantly less recurrence; and (iii) compared with PEID, PETD required a longer operative time, but was associated with less blood loss, a shorter incision, and a shorter hospital stay. Overall, PETD was better and safer than PEID.

PELD has become a more popular treatment for LDH than open discectomy. A previous study assessed the efficacy of PELD using transforaminal and interlaminar approaches [10]. However, that study had several limitations. First, only nine studies involving 621 patients were included in the analysis; we included 26 studies with 3294 patients. Second, no more than five studies were included in certain comparisons. Blood loss, bed time after surgery, and duration of hospital stay were reported in only two articles; the veracity of the pooled results is thus debatable. Third, our study involved the analysis of more information. We evaluated the short- and long-term VAS and ODI scores, and the complication and recurrence rates. Finally, our study (with a larger sample) indicated that PETD significantly reduced the blood loss, operative duration, length of incision, and duration of hospital stay; such conclusions could not be drawn in the previous study [10].

We found that PETD significantly reduced the short- and long-term VAS scores. The short-term VAS score reflects not only made improvements in disc herniation, but also the extent of surgical trauma. The incision at the intervertebral foramen was smaller (generally approximately 0.8 cm) in PETD than in PEID [19]. The PETD approach channel is expanded via blunt muscle separation, which damages tissue and muscle to lesser extents than the PEID approach [20]. Patients can feel nerve root pain during surgery. The long-term VAS scores further suggested that PETD caused less tissue injury than PEID. However, we found that PETD required a longer operative time. In general, longer spinal surgery times are associated with more complications and re-operations; surgical time is an important comparative parameter when selecting an approach. Most herniations were located at L5/S1 and L4/5, where the intervertebral disc spaces are wide; traditional surgery is not difficult. However, the anatomical structure renders it challenging to puncture and remove disc fragments via PETD, especially at L5/S1 [21,22]. Moreover, PEID is easier to surgically master; this approach uses elements of traditional surgery. We found no significant difference in complication rates, but PETD was associated with a higher recurrence rate than PEID. In terms of radiation exposure during surgery, a prospective study showed that a surgeon should perform no more than 291 procedures [23]. PETD is associated with more radiation exposure than PEID [24], reflecting the longer operative time caused by puncture difficulties, particularly in patients with high cristae iliacae, narrow foramina, or large facet joints. Radiation exposure increases with the operative time.

We found no significant difference in the complication rates of the two groups, in contrast with previous reports [10]. A retrospective cohort study including 5338 patients showed that the adult spinal surgery time was associated with several postoperative complications, including (but not limited to) wound and pulmonary complications, venous thromboembolism, the need for postoperative transfusion, length of hospital stay ≥5 days, sepsis, the need for re-operation, and unplanned re-admission [25]. We analyzed the recurrence rate, blood loss, and duration of hospital stay. These results also differed from previous findings. PETD was associated with a longer operative time, but less blood loss and a shorter hospital stay, than was PEID. We speculate that complications tend to increase with longer trauma duration; less trauma leads to fewer complications. However, the degree of heterogeneity amongst studies was high; the results may be unreliable.

The principal strength of our study was that we adhered to the PRISMA checklist and the recommendations of the Cochrane collaboration [26]. We reviewed many studies with large samples. However, limitations remain. First, most included studies were retrospective in nature; only eight were randomized controlled trials (which yield higher quality evidence). Further work is required. Second, the degree of within-study heterogeneity was rather high for certain parameters; such heterogeneity was statistical and/or clinical, and may compromise the reliability of our pooled data. Third, the surgical approach was probably influenced by disease severity/type. However, our examination of a large sample may overcome these limitations. In conclusion, PETD more effectively treated LDH than PEID. The PETD operative time was longer than that of PEID, but the two techniques were equally safe. PETD was associated with less blood loss, a shorter hospital stay, and a smaller incision than PEID. PETD should thus be preferred when treating LDH. Randomized controlled trials with larger samples are required to confirm our findings.

## Supporting information

**Supplementary Figure 1:** 

## Abbreviations

CI: confidence interval
LDH: lumbar disc herniation
ODI: Oswestry disability index
PEID: percutaneous endoscopic interlaminar discectomy
PELD: percutaneous endoscopy lumbar discectomy
PETD: percutaneous endoscopic transforaminal discectomy
RR: relative risk
SMD: standardized mean difference
VAS: visual analog scale
