# Supplementary material for: Percutaneous endoscopic transforaminal discectomy precedes interlaminar discectomy in the efficacy and safety for lumbar disc herniation
Source: Biosci Rep. 2019 Feb 15;39(2):BSR20181866. doi: 10.1042/BSR20181866 (PMC6379230; doi:10.1042/BSR20181866)
Supplement: Supplementary file 1 [file bsr-39-bsr20181866_Supp1.pdf]

Appendix 1

1.Ceng J, Tang J, Li F, et al. The comparison of curative effect between percutaneous transforaminal endoscopic discectomy and microendoscopic discectomy in lumbar disc herniation. *Chin J Prac Med* 2015; 42:79-80.

2.Chen G, Wei J. Pedicle screw for lumbar disc herniation under transforaminal endoscopy. *J Clini Reha Tiss Engineering Rese* 2015; 19:5641-5.

3.Chen Q, Qin L, Li M, et al. Comparison of the therapeutic effect of percutaneous transforaminal endoscopic discectomy and posterior discectomy on senile single segmental lumbar disc herniation. *Chin J Frontier Med Scie* 2018; 10:60-4.

4.Chen Z, Zhang L, Dong J, et al. Percutaneous transforaminal endoscopic discectomy compared with microendoscopic discectomy for lumbar disc herniation: 1-year results of an ongoing randomized controlled trial. *J Neurosurg-Spine* 2018; 28:300-10.

5.Ding Y, Hu J, Zhou Y. The effect of the descending tessys technique and disc lens on the treatment of lumbar disc herniation was compared. *J Cervicodynia Lumb* 2017; 38:492-3.

6.Fang G, Luo D, Lei G, et al. Comparison of the effect of posterior transcutaneous endoscopic and intervertebral disc endoscopic medullary nucleus pulpotomy. *Chin J Bone Joint* 2012; 27:445-6.

7.Fu Q, Gu G, Zhang H, et al. The therapy strategy of L5/ S1 lumbar disc herniation. *J Spinal Surgery* 2014; 12:44-6.

8.Guan G, Wang Y, Guan Y. Percutaneous transforaminal endoscopic discectomy

technology to treat 35 cases of lumbar disc herniation. *Contemp Med* 2014; 20:38-9.

9.Huang Z, Zhang L, Zhang Z, et al. The effective of percutaneous endoscopic lumbar discectomy for L5/S1 disc herniation via an interlaminar approach versus a transforaminal approach. *J Chin Phys* 2018; 20:507-10.

10.Le J, Du Y, Liang J, et al. The curative effect of TESSYS technology in lumbar disc herniation. *Inter Med&Health Guidance News* 2014; 20:38-40.

11. Li M, Yang H, Yang Q. Full-Endoscopic Technique Discectomy Versus Microendoscopic Discectomy for the Surgical Treatment of Lumbar Disc Herniation. *Pain Physician* 2015; 18:359-63.

12. Li Y. The curative effect observation of percutaneous minimally invasive techniques for the treatment of lumbar disc herniation. *Henan Med Rese* 2015; 24:71-2.

13. Li Z. Endoscopic protrusion removal through percutaneous intervertebral foramina to treat lubar intervertebral disc protrusion. *J Shandong Med College* 2013; 35:325-7.

14. Liu N, Chen Q, Zhong H, et al. Comparison between intervertebral foramen and intervertebral disc herniation in senile patients with lumbar disc herniation. *J Prac Orthopedics* 2017; 12:342-6.

15. Liu T, Zhou Y, Wang J, et al. Clinical efficacy of three different minimally invasive procedures for far lateral lumbar disc herniation. *Chinese Med J-Peking* 2012; 125:1082-8.

16. Liu X, Yuan S, Tian Y, et al. Comparison of percutaneous endoscopic

transforaminal discectomy, microendoscopic discectomy, and microdiscectomy for symptomatic lumbar disc herniation: minimum 2-year follow-up results. *J Neurosurg-Spine* 2018; 28:317-25.

17. Sinkemani A, Hong X, Gao ZX, et al. Outcomes of Microendoscopic Discectomy and Percutaneous Transforaminal Endoscopic Discectomy for the Treatment of Lumbar Disc Herniation: A Comparative Retrospective Study. *Asian Spine J* 2015; 9:833-40.

18. Tang M, Gong F, Wang T, et al. The comparison of curative effect between percutaneous transforaminal endoscopic discectomy
and microendoscopic discectomy. *World Latest Med Infor* 2015; 15:54.

19. Wu G, Chen C. The comparison of curative effect between percutaneous transforaminal endoscopic discectomy and
microendoscopic discectomy in lumbar disc herniation. *Contemporary Med* 2015; 21:55-6.

20. Wu HY. Controlled clinical studie of micro endoscopic discectomy combined with fiber ring suture and percutaneous endoscopic lumbar discectomy in treatment of single lumbar disc herniation
: Ninbo University, 2017:36.

21. Wu X, Zhou Y, Li Z. Percutaneous transforaminal endoscopic discectomy versus microendoscopic discectomy for lumbar disc herniation: a prospective randomized controlled study. *J Third Mili Med Univ* 2009; 31:843-6.

22. Yang L, Liao X, Zhao X, et al. Comparison of surgical outcomes between

percutaneous transforaminal endoscopic discectomy and micro-endoscopic discectomy for lumbar disc herniation. *China J Endoscopy* 2015; 9:962-5.

23. Yoon SM, Ahn SS, Kim KH, et al. Comparative Study of the Outcomes of Percutaneous Endoscopic Lumbar Discectomy and Microscopic Lumbar Discectomy Using the Tubular Retractor System Based on the VAS, ODI, and SF-36. *Korean J Spine* 2012; 9:215-22.

24. Zhang Z, Chen B. Spinal endoscope versus Fenestration laminectomy in lumbar disc herniation: a clinical comparative study. *World Clin Med* 2015; 9:86-7.

25. Zhang Z, Wang FD, Chen Y, et al. Comparison of two percutaneous endoscopic lumbar discectomy through trans-interlaminar approach in the treatment of L5/S1 lumbar disc herniation. *China J Endosopy* 2015; 21:706-9.

26. Zhao W, Li Z, Zhou Y, et al. Surgical treatment of the lumbar disc herniated discussing transforaminal endoscopic surgery system. *Orthopedic J China* 2012; 20:1191-5.

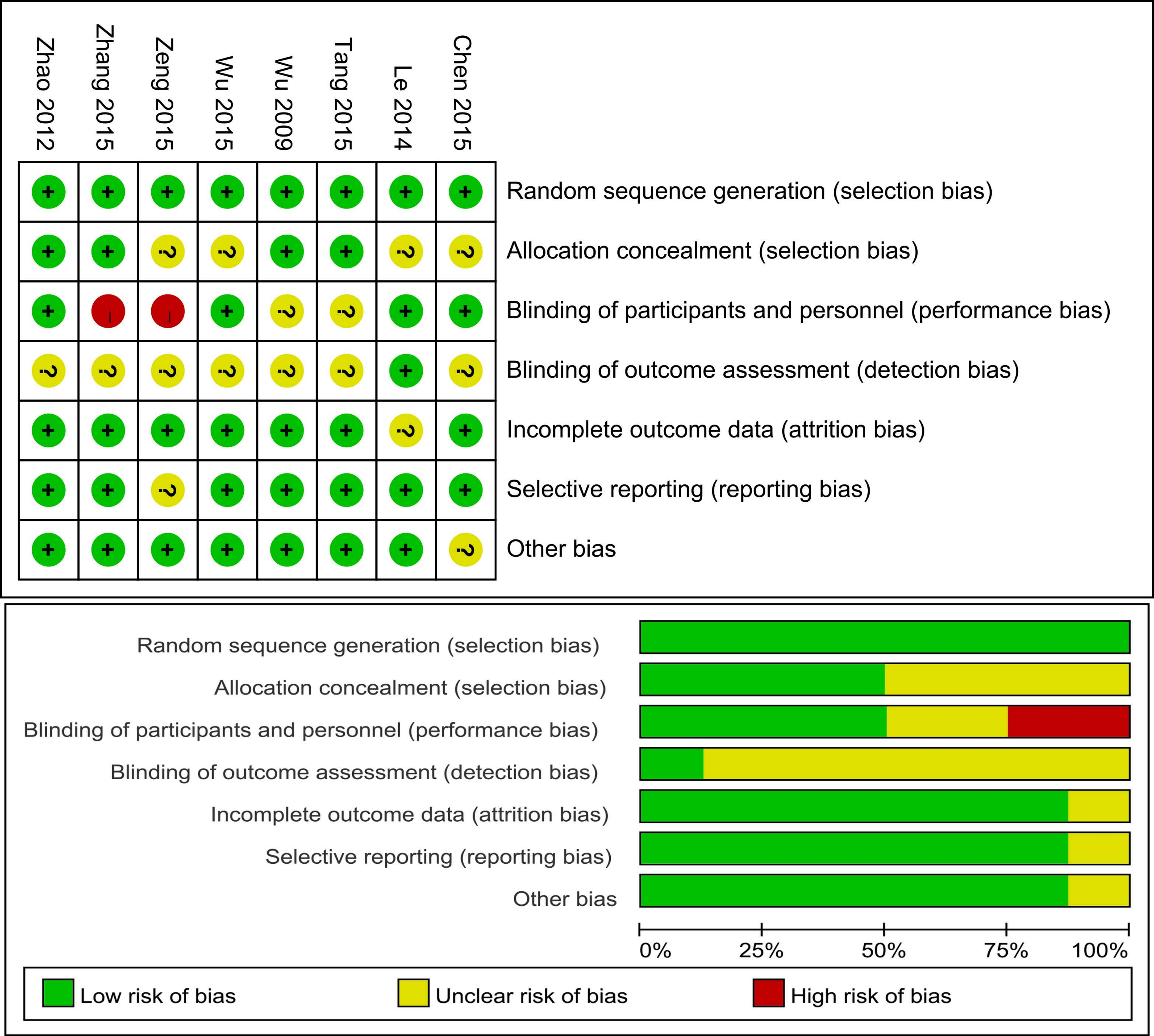

Zhao 2012
Zhang 2015
Zeng 2015
Wu 2015
Wu 2009
Tang 2015
Le 2014
Chen 2015
Random sequence generation (selection bias)
Allocation concealment (selection bias)
Blinding of participants and personnel (performance bias)
Blinding of outcome assessment (detection bias)
Incomplete outcome data (attrition bias)
Selective reporting (reporting bias)
Other bias
0%
25%
50%
75%
100%
Low risk of bias
Unclear risk of bias
High risk of bias

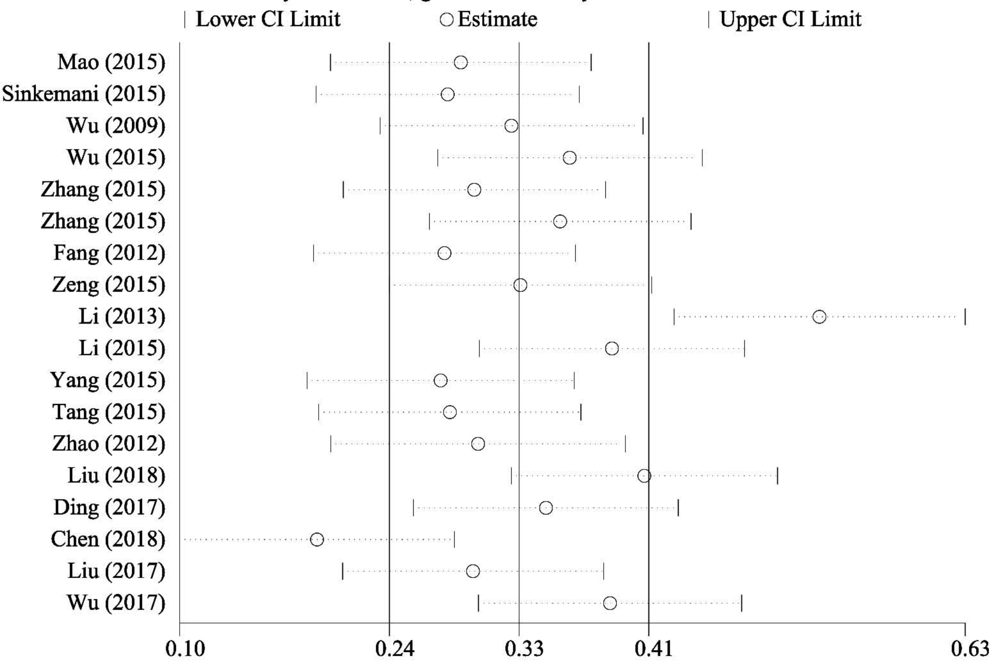

Lower CI Limit
Estimate
Upper CI Limit
Mao (2015)
Sinkemani (2015)
Wu (2009)
Wu (2015)
Zhang (2015)
Zhang (2015)
Fang (2012)
Zeng (2015)
Li (2013)
Li (2015)
Yang (2015)
Tang (2015)
Zhao (2012)
Liu (2018)
Ding (2017)
Chen (2018)
Liu (2017)
Wu (2017)
0.10
0.24
0.33
0.41
0.63

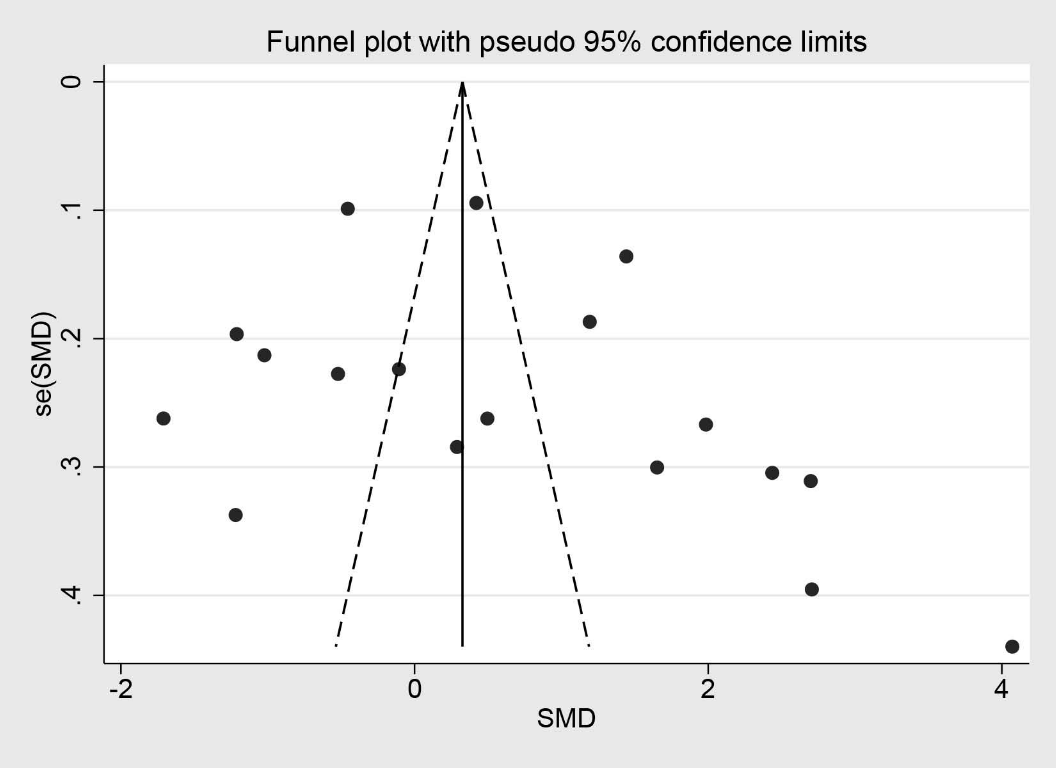

Funnel plot with pseudo 95% confidence limits
se(SMD)
0
.1
.2
.3
.4
-2
0
2
4
SMD
